# The Geographical Distribution of Probable COVID-19 Patients Transferred by Tehran Emergency Medical Services; a Cross Sectional Study 

**DOI:** 10.22037/aaem.v9i1.1177

**Published:** 2021-03-10

**Authors:** Peyman Saberian, Hosein Rafiemanesh, Mostafa Sadeghi, Parisa Hasani-Sharamin, Fatemeh Dadashi, Alireza Baratloo

**Affiliations:** 1 Prehospital and Hospital Emergency Research Center, Tehran University of Medical Sciences, Tehran, Iran.; 2 Department of Anesthesiology, Imam Khomeini Hospital Complex, Tehran University of Medical Sciences, Tehran, Iran.; 3 Department of Epidemiology, School of Public Health and Safety, Shahid Beheshti University of Medical Sciences, Tehran, Iran.; 4 Department of Anesthesiology and Critical Care, Shariati Hospital, Tehran University of Medical Sciences, Tehran, Iran.; 5 Tehran Emergency Medical Service Center, Tehran, Iran.; 6 Department of Emergency Medicine, Sina Hospital, Tehran University of Medical Sciences, Tehran, Iran.

**Keywords:** Emergency Medical Services, Geographic Information Systems, COVID-19, Tehran, Iran

## Abstract

**Introduction:**

Mapping of COVID-19 infection in the city can help us know more about how the disease is distributed and spread. This study was conducted to investigate the geographical distribution of probable COVID-19 patients who were transferred to destination hospitals by emergency medical services (EMS) in the first wave of the epidemic, in Tehran, Iran.

**Methods:**

This cross-sectional study was performed based on recorded missions during the first 3-month period of the pandemic in Tehran, Iran. All probable cases of COVID-19 who were transferred to the hospitals following contact with Tehran EMS during the study period were enrolled. Arc-GIS software was utilized to draw the distribution map of the contact places of the cases.

**Results:**

In this study, the data of 4018 patients were analyzed (60.9% male). The mean age of the patients was 54.1 ± 20.7 years; and the mean age of the patients had increased with time during the studied 3 months (p = 0.003). The average incidence rate of this disease in Tehran during the study period was 4.6 per 10,000 population. Generally, the lowest and highest raw frequencies of Tehran COVID-19 contamination were seen in municipal districts 21 and 4, respectively. The raw frequency of cases during the 3-month study period also showed that the highest number of cases in Tehran occurred in municipal districts 5 and 4, respectively.

**Conclusion:**

In the present study, using geographic information systems (GIS), geographical distribution map of COVID-19 in Tehran, Iran, during the first 3 months of the pandemic was drawn.

## Introduction

 Since the outbreak of a new coronavirus, SARS-CoV-2, various studies have been designed and performed to assess different dimensions of its resulting disease, named COVID-19 (1). Certainly, the most important part of these studies is devoted to finding a treatment or vaccination, however, other aspects of this pandemic are also of particular importance and require research. One of the important aspects of this pandemic may be the epidemiological aspect of the disease and how the epidemic spreads geographically (2, 3). Geographic information system (GIS) is one of the tools used to evaluate such information, which can show the patterns of epidemic progression in geographical units (4). Indeed, health professionals have long considered conventional mapping and more recently the GIS, as critical tools in tracking and combating contagion. Researchers have stated that, like in the previous epidemic of SARS-CoV in 2003-2004, seasonal flu, and etc., GIS can be very helpful in the current pandemic and provide a lot of information at different levels to its users. For example, online mapping of infected cases in the city can help to know more about how the disease is distributed and spread. There is also a wide range of GIS applications for mapping and tracking the SARS-CoV-2 epidemic and its related events (5). The world health organization (WHO) has put monitoring of the disease around the world on the agenda to provide a clear vision of the pandemic. This vision contains information on the overall status of patients, involved countries, the number of deaths, and countries with the highest rate of mortality. Such information can provide an overview of the disease for health policymakers, researchers, and people around the world to make appropriate decisions based on the circumstances (6). Considering the above reasons, this study was conducted to investigate the geographical distribution of probable COVID-19 patients who were transferred to destination hospitals by Tehran emergency medical services (EMS) in the first phase of the epidemic and describe how the disease spread geographically in Tehran in the first phase.

## Methods

***Study design***

This cross-sectional study was conducted in Tehran, Iran. It was performed using the previously recorded information in the registry system of Tehran EMS in a 3-month period from February 20, 2020, to May 20, 2020. First month was Esfand,1398 (in solar calendar) compatible with February 20, 2020-March 19, 2020; 2^nd^ month was Farvardin, 1399 (in solar calendar) compatible with March 20, 2020-April 19, 2020; and 3^rd^ month was Ordibehesht, 1399 (in solar calendar) compatible with April 20, 2020-May 20, 2020.

In order to conduct the study, the necessary permission to access the information was obtained from Tehran EMS center and also the study proposal was approved by the ethics committee of Tehran University of Medical Sciences (code: IR.TUMS.MEDICINE.REC.1399.413). Confidentiality of information was maintained and therefore all information was recorded, analyzed, and reported anonymously.

***Study population***

Census method was used for sampling and the study population in this study included all probable cases of COVID-19 (based on WHO definition (7)) who were transferred to the hospitals following contact with the EMS during the study period and their information was recorded in the registry system. If the patient's file was incomplete or the patient had died before the emergency technicians arrived, the case was excluded. Also, patients with other complaints who were transferred to the hospital by the Tehran EMS, and were diagnosed with COVID-19 during the diagnostic procedures were not included.

**Figure 1:**
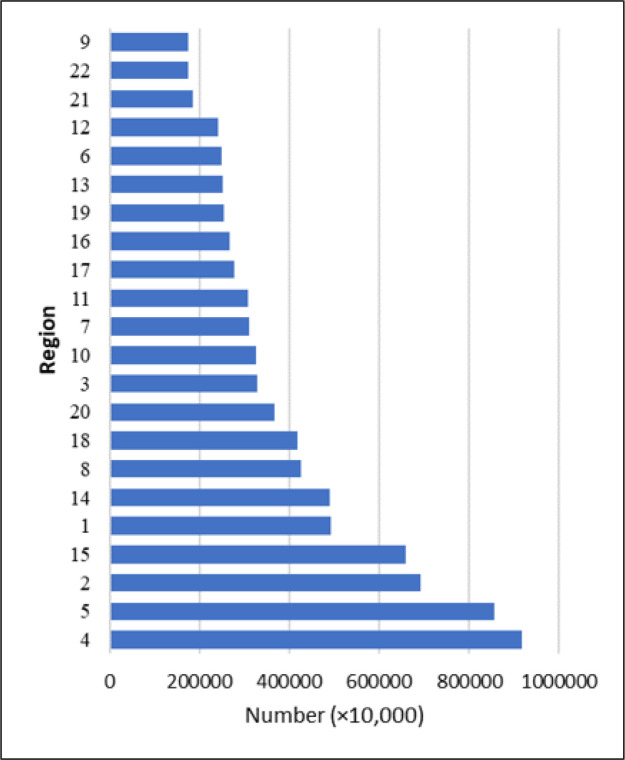
Distribution of the population in 22 districts of Tehran based on the 2016 census

***Definitions*** According to the 2016 census, Tehran has a population of 8,680,000, with district 9 and district 4 being the least and the most populated geographical districts among the 22 municipal districts, respectively (Figure 1). In order to cover this population over the area of 730 square kilometers in Tehran, Tehran EMS center had 217 stations and 250 ambulances in the city, and 2200 technicians throughout the duration of this study. According to the policies adopted by the Ministry of Health and Medical Education following the COVID-19 epidemic, there was a strong recommendation that patients should be transferred by the EMS, and it was frequently announced through the public media, and therefore, all patients quarantined at home after the diagnosis was confirmed, contacted the EMS as soon as symptoms worsened or a new complaint was raised. At first, these patients were transferred only to a limited number of medical centers (n=10) that were allocated to these patients, but later, following the change of instructions by the Iranian Ministry of Health, they were transferred to all general medical centers in the city. According to the statistics of the Iranian ministry of health and medical education, the number of patients with COVID-19 who were transferred to hospitals was about 22% of the total number of infected cases in Tehran.

***Data gathering***

The data were extracted from patients' files and Tehran EMS registry system using a researcher-made checklist. This checklist consisted of two main parts: the first part included the demographic data and information related to the disease (age and sex); The second part included the temporal-spatial information of the missions (the caller’s location at the time of contact with the EMS dispatcher).

***Statistical analysis***

In this study, the number of probable COVID-19 cases was calculated and presented as the raw frequency and the monthly incidence rate for the population of each district. To calculate the incidence rate based on the population as well as other population indicators such as population per hectare and working population, the census statistics of 2016 were used. Arc-GIS software was utilized to draw the distribution map of the cases. Also, ANOVA test was used to examine the relationship between quantitative variables (such as mean age) in the 3-month study period, and the Pearson Correlation was used to examine the ecological correlation between the number of cases with the population per hectare of each district and the working population of districts. Analytical analyzes were performed at a confidence level of 95% using Stata software version 14.

## Results

In this study, the data of 4018 patients with a probable diagnosis of COVID-19 in Tehran (divided into 22 districts) during the first three months of the COVID-19 epidemic were studied (60.9% male; male/female ratio = 1.6). The frequency of patients in different age groups by sex is reported in table 1.

**Figure 2:**
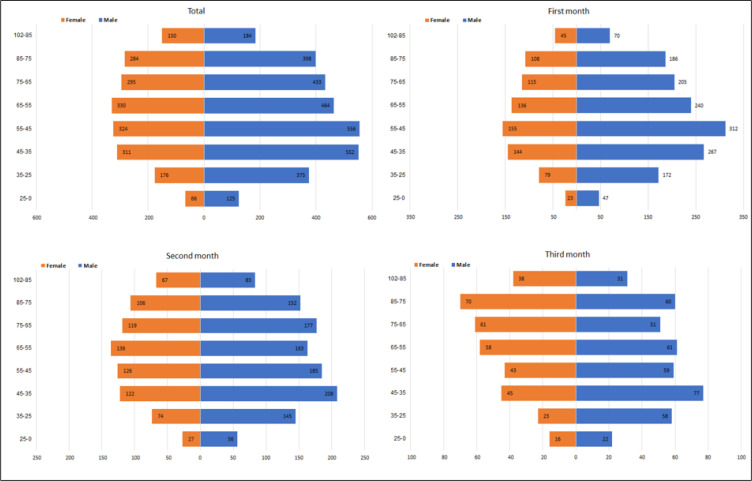
The pyramid of the age-sex frequency of missions in Tehran (22 districts) during the first three months of the COVID-19 epidemic

The mean age of the studied patients was 54.1 years (SD = 20.7). The pyramid of the age-sex frequency during the first three months of the COVID-19 epidemic is shown in figure 2. In general, the number of patients, both male and female, was lower in the age group <35 years. The mean age of the studied patients in the 1^st^, 2^nd^ and 3^rd^ month of the study was 53.2 (SD = 20.1), 54.5 (SD = 21.0), and 56.0 (SD = 21.8) years, respectively (p = 0.003).

**Figure 3:**
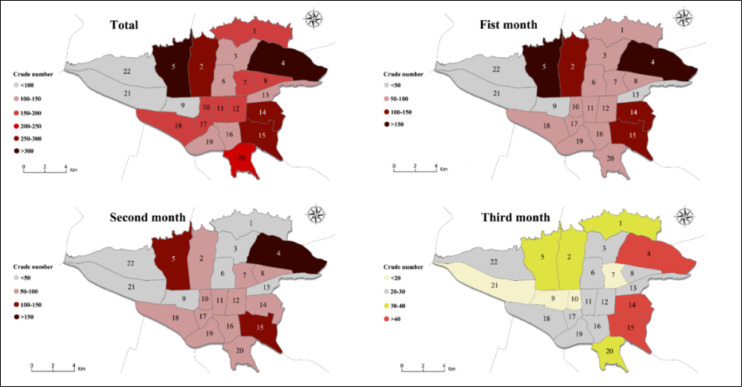
Geographical distribution of the crude number of COVID-19 patients during the first three months of the COVID-19 epidemic in Tehran

The geographical distribution of the studied patients in different months is shown in figure 3. Generally, the lowest and highest raw frequencies of Tehran COVID-19 contamination were seen in municipal districts 21 and 4, respectively. The districts 4 and 5 accounted for 18.6% of the total infected cases. In all districts, the number of cases was higher for males than for females, and in districts 12, 17 and 21, the number of infected males was more than twice the females.

The raw frequency of cases in the 3 studied months also showed that the highest number of cases in Tehran occurred in municipal districts 5 and 4, respectively. In 1^st^ month, the number of cases had reached less than 100 in all districts, most of which were seen in the districts 4, 14, and 15 with 46, 47, and 63 cases, respectively. Also, the overall number of male cases in all districts was higher than female cases during the three studied months. However, the difference between the number of male and female cases decreased in the 3^rd^ month and in some districts (2, 3, 6, 7, 16, 19, and 22) the number of contaminations in females was higher than males. The number of cases in most districts of Tehran decreased from the 1^st^ to the 3^rd^ month, and this decrease was greater for districts with more cases (such as district 4 and 5). In District 22, the changes during the 3 months of the study were not significant.

**Incidence rate based on population**

**Figure 4:**
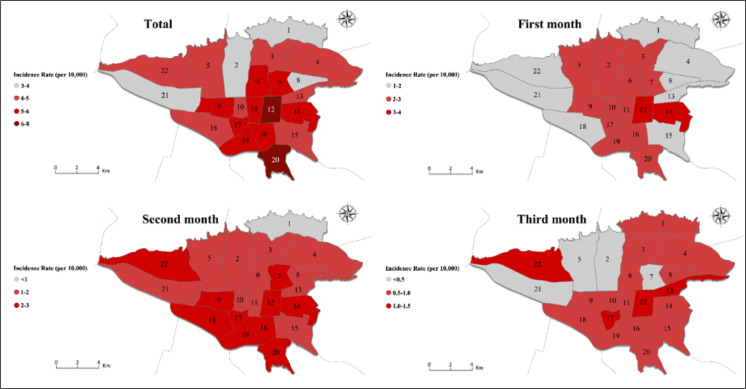
Geographical distribution of incidence rate (per 10,000 population) of COVID-19 during the first three months of the COVID-19 epidemic in Tehran

In total, for the whole of Tehran, the incidence rate of COVID-19 during the 3 studied months was 4.6 cases per 10,000 population. In the 1^st^, 2^nd^, and 3^rd^ month, this rate was 2.16, 1.78, and 0.70 per 10,000 population, respectively. The lowest and highest incidence rates in Tehran during the studied 3 months were in the municipal districts 21 and 12 with 3.27 and 7.18 per 10,000 population, respectively. In district 12, incidence rate in the 1^st^ month and the 2^nd^ month was 3.45 and 2.53 per 10,000 population, respectively. In the 2^nd^ month, district 22 had the highest monthly incidence rate (1.43 per 10,000 population; Figure 4).

**Figure 5:**
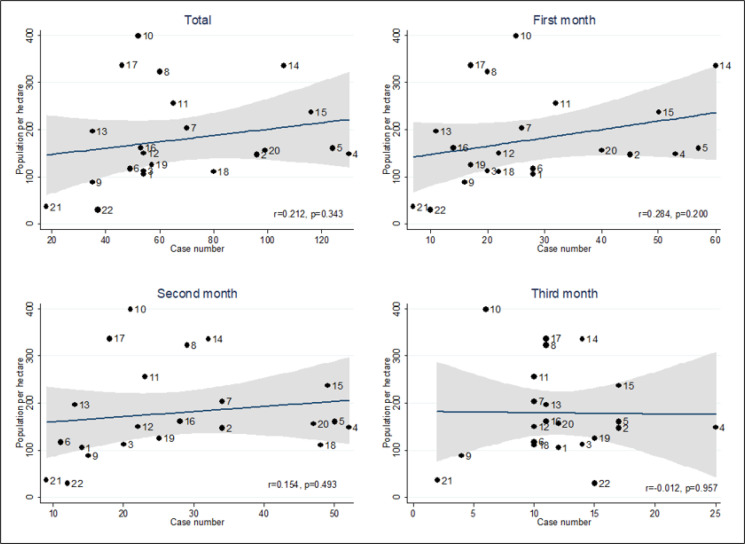
Correlation between population per hectare in the districts of Tehran and the number of missions during the first three months of the COVID-19 epidemic

**Figure 6:**
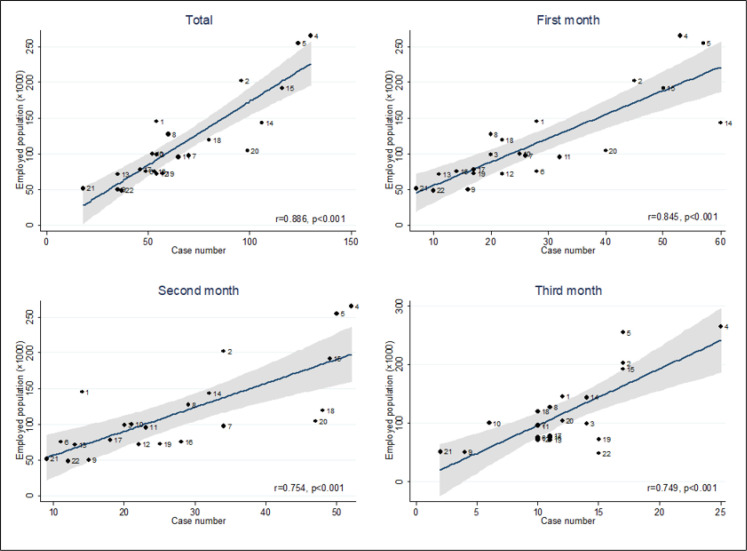
Correlation between the employed population in each district of Tehran with the number of missions during the first three months of the COVID-19 epidemic

During the 3 studied months, the incidence rate in males and females was 5.67 and 3.41 cases per 10,000 population, respectively. The monthly incidence rate for males in 1^st^, 2^nd^, and 3^rd^ month was 2.79, 2.10, and 0.77 per 10,000 males, respectively, and for females these rates were 1.42, 1.39, and 1.60 per 10,000 females, respectively. There was no significant relationship between the population per hectare in each district with the number of cases, although this correlation was generally positive, and in districts with a higher population per hectare the number of cases was higher (r = 0.212, p = 0.343) (Figure 5). There was a significant relationship between the working population of each district with the number of cases (r = 0.886, p <0.001), this strong significant positive correlation was also seen in each of the 3 months of study separately (Figure 6).

## Discussion

In the present study, it was found that based on the probable cases registered in Tehran EMS Center, the average incidence rate of this disease in Tehran was 4.6 per 10,000 population during the study period, and the lowest and highest incidence rates belonged to the municipal districts 21 and 12, respectively. Although in this study the correlation between population per hectare in each district and the number of cases was generally positive, and the districts with a higher population had more cases, no statistically significant relationship was found between them.

To the best of our knowledge, there is no similar article, conducted on the same population, because no one else has access to this information, which was extracted from Tehran EMS registry bank. There is one that was performed to give a spatial analysis of COVID-19 spread from February 19^th^ to March 18^th^, at a province level in Iran, which is interestingly conducted by Ramírez-Aldana et al. (8), who are Mexican authors! However, they found that there was a considerably higher density of COVID-19 patients around Tehran province. Hazbavi et al. assessed COVID-19 incidence pattern, using geo-database, during seven successive periods in Iran, again at province level. They found that in the second month of the pandemic (February 18–March 19 of 2020), Tehran province, with more than 1000 infected cases, was one of the highly affected provinces of Iran. Tehran had the most deaths with an increasing trend for all the study periods (9). These findings confirm the special importance of the study of Tehran in this regard.

During the period of the present study, the highest number of patients with COVID-19 were transferred to hospitals by EMS system from districts 4 and 5 of Tehran. Reviewing the maps, it can be also seen that the incidence of this disease in the third month of the study, which was in fact 3 months after the official announcement of the first case in Iran, had decreased significantly. Also, districts 21, 22, and 9 of Tehran had the lowest number of cases throughout the study, which is consistent with the population of the districts. In other words, districts with lower population had always accounted for a smaller number of cases. Moreover, 4 densely populated districts of Tehran, including districts 4, 5, 2, and 15 always had the highest number of cases. Analysis of incidence rate based on the population showed that the spatial changes of the epidemic started from the central districts of the city, and in the second and third months it expanded throughout Tehran, especially to the districts that were not involved. Meanwhile, districts such as 1, which is considered as one of the districts with a high level of socio-economic status, always had a lower rate of incidence for the population; so that in the first and second months it had the least number of infections, and in the third month the number of cases was between 0.5 to 1 per 10,000 population. The same pattern can be seen in other districts with higher socio-economic status like districts 2 and 5. This finding suggests that socioeconomic factors may also be influential in the speed of COVID-19 spread. In contrast, some central districts of Tehran, such as district 12, had the highest number of cases, which can be attributed to having the greatest number of scientific, commercial, etc. centers, and traffic. However, the central parts of Tehran, which usually have the highest population density during working hours, such as districts 6, 10, 11, and 12 were in a better situation than expected, which may be the result of shutting down of businesses and markets, and traffic reduction in these particular districts. Besides, it should be acknowledged that many businessmen and people who commute to the mentioned districts do not inhabit there and travel from other areas of Tehran or even the suburbs of Tehran.

Although infected cases were more commonly >35 years old, the disease was found in all age groups. The mean age of patients during the first three months of the COVID-19 epidemic was increasing with time and the difference between the mean age of patients between the 1^st^ and 3^rd^ month was statistically significant. This could be due to a change in the age pattern of infected cases, assuming the call pattern has not changed during the 3 months. Table 1 and Figure 1 demonstrate that the age-sex pattern of missions has changed from the young to the elderly and from male to female during the 3 months of the study.

In this study, no significant correlation was found between population density, based on population per hectare, of each district and the number of cases. Probably one of the reasons for this finding is the lower number of residential use; for example, although districts 21 and 22 are larger, they do not necessarily have a lower population density, and they are probably not much different from smaller districts in terms of population density in residential areas. The additional space of larger districts can be attributed to non-residential areas that have no effect on population density.

The results of this study showed a strong positive correlation between the number of the working population in each district and the number of cases. This correlation was seen in the overall duration of the study as well as for each month of the study, separately. This finding shows that the working population can be one of the most important factors in the spread of the COVID in society. It suggests that one of the ways to control the epidemic is by paying attention to the working population and develop preventive protocols for this population.

Considering the fact that Tehran EMS is a concentrated system and is responsible for providing services to the entire metropolis of Tehran, by developing appropriate policies to create online and instant management dashboards and using the calls and missions of this center, the spread of the epidemic can be assessed in different districts and the necessary preventive strategies to control and restrain the spread of the virus can be implemented.

Once the situation in different districts of Tehran is determined in terms of the number of inhabitant cases in each district, it is time to identify and analyze the factors that caused these fundamental and tangible differences by planning appropriate studies.

**Table 1:** Frequency of each age group in males and females in each of the three studied months and the overall period of the study

**Age group**	**First month**		**Second month**		**Third month**		**Total**	
	**Male, n=1499 **	**Female, n=805 **	**Male, n=1169 **	**Female, n=777 **	**Male, n=419 **	**Female, n=354 **	**Male, n=3087 **	**Female, n=1936 **
0-25	47 (3.1)	23 (2.9)	56 (4.8)	27 (3.5)	22 (5.3)	16 (4.6)	125 (4.0)	66 (3.4)
25-35	172 (11.5)	79 (9.8)	145 (12.4)	74 (9.5)	58 (13.8)	23 (6.7)	375 (12.1)	176 (9.1)
35-45	267 (17.8)	144 (17.9)	208 (17.8)	122 (15.7)	77 (18.4)	45 (13.0)	552 (17.9)	311 (16.1)
45-55	312 (20.8)	155 (19.3)	185 (15.8)	126 (16.2)	59 (14.1)	43 (12.5)	556 (18.0)	324 (16.7)
55-65	240 (16.0)	136 (16.9)	163 (13.9)	136 (17.5)	61 (14.6)	58 (16.8)	464 (15.0)	330 (17.0)
65-75	205 (13.7)	115 (14.3)	177 (15.1)	119 (15.3)	51 (12.2)	61 (17.7)	433 (14.0)	295 (15.2)
75-85	186 (12.4)	108 (13.4)	152 (13.0)	106 (13.6)	60 (14.3)	70 (20.3)	398 (12.9)	284 (14.7)
85-102	70 (4.7)	45 (5.6)	83 (7.1)	67 (8.6)	31 (7.4)	38 (11.0)	184 (6.0)	150 (7.7)
Mean±SD	54.3±18.1	55.5±18.3	54.8±19.8	57.2±19.0	54.3±20.3	60.8±19.8	54.5±19.1	57.1±19.0

Data are presented as frequency (%). SD: standard deviation.

## Limitations

The database is based on cases transferred by the EMS so it does not include all diagnosed cases because some people went to hospitals and medical centers without contacting EMS, whose data was not included. Moreover, cases who were transferred to the hospital by the EMS with other chief complaints and were diagnosed with COVID-19 during the diagnostic procedures were not included in the study. On the other hand, those who were transferred as probable COVID-19 cases but the disease was subsequently ruled out were not identified.

## Conclusion:

In the present study, using GIS, geographical distribution map of COVID-19 in Tehran, Iran, during the first 3 months of the pandemic was drawn. The mapping can help compare the 22 municipal districts of Tehran in this regard and hopefully be suitable for use by governmental health managers.

## Competing Interests

None.

## Funding

This study was funded with a grant from Tehran EMS Center.

## Authors’ contribution

The conception and design of the work by PS, PHS, MS and AA; Data acquisition by PHS, HR and FD; Analysis and interpretation of data by HR and AA; Drafting the work by HR, PHS, FD and AA; Revising it critically for important intellectual content by PS and MS; All the authors approved the final version to be published; AND agree to be accountable for all aspects of the work, ensuring that questions related to the accuracy or integrity of any part of the work will be addressed.
